# Cardiorespiratory fitness is associated with cognitive performance in 80 + -year-olds: Detangling processing levels

**DOI:** 10.1007/s11357-024-01065-8

**Published:** 2024-01-23

**Authors:** Stephanie Fröhlich, Dieter F. Kutz, Katrin Müller, Claudia Voelcker-Rehage

**Affiliations:** 1 Department of Neuromotor Behavior and Exercise, University of Münster, Wilhelm-Schickard-Str. 8, 48149 Münster, Germany; 2 Institute of Human Movement Science and Health, Chemnitz University of Technology, Chemnitz, Germany

**Keywords:** Cardiorespiratory fitness, Cardiovascular fitness, Executive function, Selective attention, Inhibitory control, Flanker task, Older adults, Event-related potentials, Motor-related cortical potentials

## Abstract

Cardiorespiratory fitness is known to protect against cognitive decline in older adults. Specifically, it has been shown that physical activity and fitness are beneficial for executive functions that are crucial for independent living up to old age. In this study, 115 individuals aged 80 years and older underwent a cardiorespiratory fitness assessment using the two-minute step test and had their electroencephalogram recorded during a colored flanker task in order to measure executive function performance. Cardiorespiratory fitness was related to quicker responses during the flanker task. A mediation analysis was carried out to determine whether these positive effects were mediated through event-related potentials (N1, N2, or P3) or motor-related cortical potentials (MRCP). Cardiorespiratory fitness was related to better visual discriminative processing as indicated by larger occipital N1 amplitudes. In addition, fitness was associated with larger MRCP amplitudes, which are a correlate of the response generation process. Fitness was not found to have a significant effect on fronto-central N2 or parietal P3, which are thought to capture cognitive control processes such as conflict detection and response inhibition. Moreover, all effects reported were present in all three flanker trial conditions (congruent, neutral, and incongruent). Thus, these results indicate that the quicker response times in fitter people were related to visual processing and motor response generation rather than cognitive control.

**Supplementary Information:**

The online version contains supplementary material available at 10.1007/s11357-024-01065-8.

The term executive functions (EF) refers to a range of cognitive top-down processes that are necessary for goal-oriented behavior and, subsequently, influence many aspects of our everyday lives (e.g., well-being, health, and success at work and school) [1]. Unsurprisingly, there is a clear relationship between EF and preservation of independence into old age, even in cases of mild cognitive impairment [2, 3]. Considering the growing number of older adults (OA) and the lack of resources in the health and elderly care sectors, it is important that we identify ways to support independence in old age [4]. Cardiorespiratory fitness is known to be a protective factor against cognitive decline and physically active OA have shown better cognitive performance than their less fit counterparts [5]. In cross-sectional studies various fitness parameters were correlated with various dimensions of cognition [6–8] and there is some evidence that EF are particularly sensitive to the effects of chronic physical activity and fitness [9, 10]. For example, comparisons between more and less physically active OA [11–13] and comparisons between OA with high and low cardiorespiratory fitness [14–16] have shown a clear performance advantage in EF in reaction times for the fitter, more active individuals. However, the relationship between cardiorespiratory fitness and EF has not yet been studied sufficiently in the old-old age group, over 80 years of age [17, 18]. Further, the effects of fitness are likely moderated by the age [11, 19] and cognitive status of the OA [20]. In addition, the understanding of the underlying neural processes involved in the link between fitness and EF in older adults in general and in 80 + -year-olds specifically is lacking [9, 21].

In order to better understand how the cognitive benefits of fitness arise, brain structural and functional parameters are needed. Some studies have, therefore, focused on quantifying EF performance by measuring the electroencephalogram during an EF task and calculating event related potentials (ERP) [11, 14, 15]. Two core EF subfunctions of interest in this context are attentional control, which is the ability to focus our attention on relevant stimuli and to ignore others, and response inhibition, which is the ability to inhibit automated responses [1, 22]. Both are needed to successfully perform many tasks in everyday life and can be systematically studied by the flanker task in which a target stimulus is surrounded by distracting flankers [23]. The stimulus-locked ERPs elicited by the flanker task allow conclusions to be drawn about the timing and resources of early visual processing (N1) and higher order cognitive control processes (N2, P3) related to attentional control and response inhibition [24]. Moreover, the ERPs during the flanker task show age-related changes [25] making them a suitable measure to study the effect of fitness on cognition in OA. Research so far has mainly focused on the effects of fitness on P3 in OA [19]. The P3 is related to context-updating and inhibition of task-irrelevant neural activity [26, 27]. Better fitness and elevated levels of physical activity have been associated with larger P3 amplitudes and faster latencies, which can be interpreted as greater attentional resources and faster cognitive control processes [15, 19]. However, other ERPs (such as N1 or N2) have not been studied as frequently. For example, physically active healthy OA also had enlarged N1 amplitudes, which has been interpreted as more resources available for the identification of stimuli during early visual processing [12]. Furthermore, physical activity status of middle-aged adults was related to amplitude modulations during trials with visual conflict in the N2 but not in the P3 component, which was interpreted as increased capabilities to ignore visual distracting information [11].

Successful performance in choice reaction tasks such as the flanker task also involves exhibiting a quick motor response. The motor response is, in turn, comprised of the planning, preparation and execution stages. It is well known that OA experience decrements in their motor control that are related to both, peripheral changes (neuromuscular and sensory system) and changes in the central nervous system [28]. Yet, motor planning and preparation is rarely considered, when studying the effects of fitness on cognition. Therefore, the motor related cortical potentials (MRCP) were also of interest to study the planning and preparation of the motor response [29]. It has been shown that during a choice reaction task OA have larger MRCPs with later onset compared to younger adults, this is then connected to longer reaction times (RT) [30]. A similar ERP component prior to keypress, termed N0, has also been reported on in choice reaction tasks in which it was interpreted as signifying the end of the deliberation process because the peak was earlier on the ipsilateral than the contralateral side of the hand for the keypress [31, 32]. As previously mentioned, when compared to the effects of stimulus-locked ERPs, far less is known about the relationship between fitness and MRCPs. One study has shown differences in frontal MRCP (smaller latency, smaller amplitude) in a physically active as compared to a non-active group [33]. In that study, frontal activity was measured instead of the activity of the contralateral motor area. Hence, the results were interpreted as showing beneficial effects on prefrontal cortex mediated executive functions influencing response planning and response execution [33].

The aim of this study was to further expand the understanding of the relationship between cardiorespiratory fitness and EF performance, specifically attentional control and response inhibition, in a sample of old-old (≥ 80 years) adults. In addition, this study investigated the associated underlying neural processes by including ERP parameters that measure early visual processing (N1), cognitive control (N2, P3) and motor planning (MRCP). It was hypothesized that better cardiorespiratory fitness would be associated with larger amplitudes and shorter latency in all stimulus-locked potentials. Regarding the effect of fitness on MRCPs, no clear hypothesis was proposed as we were only able to identify one previous study on this relationship [33].

## Methods

### Sample

The current sample is a subsample of participants from the SENDA study [34]. In the SENDA study, non-demented persons aged 80 years and older were recruited in Chemnitz, Germany, and its surrounds. The exclusion criteria were: (1) diagnosis of acute psychological disorders (such as major depressive disorder) (2) diagnosis of neurocognitive disorders, (3) permanent impairments due to stroke or brain surgery, (4) diagnosis of other neurological diseases (such as epilepsy, Parkinson, or neuropathy) (5) substance abuse, (6) participation in other clinical studies, (7) a physician-directed ban from physical activities, (8) no walking ability, (9) severe restrictions due to cardiovascular, pulmonary, or orthopedic diseases, and (10) failure to reach the minimum required score of 19 during screening with the Montreal Cognitive Assessment (MoCA, [35]). The study was approved by the Ethics Committee of the Chemnitz University of Technology (TU Chemnitz), Faculty of Behavioral and Social Sciences, on December 19, 2017 – number V-232–17-KM-SENDA-07112017. Informed consent was obtained from all individual participants included in that study. The trial was retrospectively registered at German Clinical Trials Register (DRKS) with registration number DRKS00013167. In this study, we analyzed a subsample of *N* = 138 cognitively healthy participants according to the MoCA cut-off criteria (MoCA ≥ 23, Carson et al. [36]), that took part in the cardiorespiratory fitness assessment and the flanker task. In addition, we also excluded participants from our data analysis due to: accuracy in the flanker task below chance overall or in at least one condition (*n* = 4), a flanker answer rate below 75% (*n* = 1), technical issues during recording (*n* = 2), the participant did not follow the instructions for the task (*n* = 1), noisy EEG data (*n* = 3), colorblindness (*n* = 2), inability to do the fitness assessment (*n* = 1), and being left-handed according to the Oldfield Handedness Inventory (*n* = 9). The final sample included *N* = 115 participants (age: *M* = 82.4 years, *SD* = 2.3) and consisted of 55 females and 60 males (see Table 1 for further demographic data).

**Table 1:** Demographic data

Variable	*M* (*SD*)
Sex (f/m)	55/60
Age (years)	82.4 (2.3)
Education (years)	14.2 (3.1)
MoCA	26.3 (1.9)
TMST	78.1 (19.3)

*MoCA* montreal cognitive assessment, *TMST* two minute step test

### Material

#### Flanker task

**Fig. 1:**
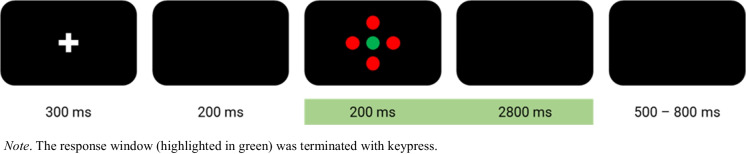
Setup of a trial in the flanker test

A modified Eriksen flanker task [23] was used to study attentional control and response inhibition. Presentation (Neurobehavioral Systems, Berkeley, CA, USA) was used to present the stimuli, record responses, and send markers to the EEG. The stimuli consisted of a center disk (17 mm) surrounded by four flanker disks of the same size displayed against a black background (Fig. 1) on a 23.8 inch monitor (hardware resolution 1920 × 1080 pixels). Participants were asked to ignore the flanker disks (blue, red, or green) and to react only to the color of the center disk (red or green). Responses were carried out by pressing the down arrow key (marked green) with the right index finger and the right arrow key (marked red) with the right middle finger of a commercial German keyboard. The trials required the resolution of different amounts of distractor conflict based on the color combination of the target and flankers. Congruent (C) trials (all green or all red) had no conflict, neutral (N) trials included only visual conflict (blue flankers around a red or green target) and incongruent (IC) trials had added response conflict (green flankers around a red target or vice versa). This colored flanker version was adapted for the sample age by increasing the stimulus size and expanding the response window used by Winneke et al. [11].

The experiment consisted of 20 practice trials and three blocks of 100 task trials. All three types of stimuli (C, N, IC) were presented randomly with equal probability. One trial (Fig. 1) consisted of a fixation cross (300 ms), a blank screen (200 ms), stimulus presentation (200 ms), a blank screen during the response interval (terminated by key press, maximal 3000 ms), and a blank break interval screen (randomly allocated between 500 to 800 ms). The whole flanker task (including practice trials) took participants approximately 12–15 min.

#### EEG Recording

We used the actiCHamp system (Brain Products GmbH, Gilching, Germany) with 32 active EEG electrodes to record the EEG during the Flanker task. The electrodes were positioned according to the international 10–20 system at Fp1, Fp2, F7, F3, Fz, F4, F8, FC5, FC3, FC1, FC2, FC4, FC6, T7, C3, Cz, C4, T8, CP5, CP3, CP1, CP2, CP4, CP6, P7, P3, Pz, P4, P8, O1, Oz, and O2. The setup included an online reference electrode at Fz and a ground electrode at Fpz. We kept electrode–skin impedance below 25 kΩ and recorded at a 500 Hz sampling rate and 24-bit resolution.

#### Cardiorespiratory fitness

The two-minute step test [TMST, 37] was used to assess cardiorespiratory fitness because it is an appropriate measure for OA and has good validity [38]. Participants raised one knee at a time, alternating between the left and right knee, and completed as many repetitions as they could in two minutes. The number of lifts completed with the right knee was recorded. The target height of each knee lift was individually marked for each participant so as to be level with the halfway point between the midpoint of their patella and the top of their iliac crest.

### Procedure

The TMST and EEG recording during the flanker task only made up a small part of the testing on the day. Further measurements taken but not included in this analysis involved other motor tasks including gait, balance, grip strength, and fine motor performance, in addition to a demographic and health questionnaire. The order of measurements was as follows: Participants were randomly allocated to perform a dual-task walking test or the same cognitive task while seated. Subsequently, a short physical performance battery was performed with the TMST as the final assessment. Following this, the unfulfilled condition of the dual-task paradigm (sitting or walking) was executed. The remaining procedural steps remained constant, with the flanker test performed following the fine motor tasks, which used the same EEG setup. Rest periods were offered throughout the procedure, and all participants were given a break prior to their EEG recording. All the tests took each participant approximately 2.5 h.

### Data analysis

#### Flanker performance

The preprocessing of the flanker RT data was done using the R base package [39]. Performance parameters (RT and accuracy) were calculated separately for each condition. Prior to averaging, RT < 100 ms were removed because it is not possible to consciously process the stimuli this quickly. In addition, trials with a RT more than two standard deviations slower than the individual mean for the trial type were also removed [40]: only 4% of the trials were removed for these reasons. Afterward, the mean RT was calculated for each trial type based on correct responses only. Table 2 presents the average performance per condition, which confirms that the manipulation of distractors was successful by showing that performance in all outcomes deteriorated with increasing distractor conflict.

**Table 2:** Behavioral data (*N* = 115) for the colored flanker task

	Congruent	Neutral	Incongruent
RT in ms *M* (*SD*)	495 (69)	508 (69)	533 (71)
Accuracy in % *M* (*SD*)	95 (4)	94 (5)	90 (7)

*RT* reaction times

#### EEG preprocessing and ERP analysis

BrainVision Analyzer 2.2.1 (BrainProducts GmbH, Gilching, Germany) was used for all preprocessing steps. The data was filtered with a zero-phase-shift Butterworth filter (low cut off 1 Hz, order 8; high cut off 30, order 8; notch filter 50 Hz). Eye blinks were corrected via the software implemented Ocular Correction ICA [41] using Fp1 as the reference channel for vertical eye movements. Afterwards, re-referencing was carried out to a common average reference including all 32 electrodes used in the setup. Only trials with a correct answer given within 1500 ms of the presentation of the stimulus were included in the following steps of the analysis. In order to be able to analyze both stimulus-locked and response-locked activity, the continuous data was segmented into 2500 ms epochs relative to the stimulus onset (-200 ms to 2300 ms). A baseline correction was carried out for each segment by subtracting the average pre-stimulus activity (-200 ms to 0 ms). Afterward, automatic electrode-wise artifact rejection was used to remove all trials in which the maximal allowed difference of 100 µV within intervals of 100 ms was exceeded [25].

Based on these pre-processed epochs, stimulus-locked ERPs were obtained by averaging the segments of each condition (C, N, IC) and automatically detecting global peaks in the following time windows: (1) N1 at O1 and O2: negative peak 100 – 200 ms, (2) N2 at Cz and Fz: negative peak 200 – 400 ms, and (3) P3 at Pz: positive peak 350 – 650 ms. These time windows were determined based on Reuter et al. [25], who used the same flanker task, with one modification: The P3 time window was adjusted to be 50 ms later to account for the sample age [42]. The automated peak analysis was checked visually and the search windows were adapted when necessary (n = 20, ca. 17% of the sample). Of the responses that were adapted, the search window for N1 was extended to 250 ms for twelve participants, the N2 window was shortened (individual windows) for four participants, and the P3 window was moved forward 50 ms to 300 – 600 ms for ten participants. Some participants’ results required multiple adaptions.

From the same pre-processed epochs segments relative to the time of keypress (-500 ms to 500 ms) were extracted. The MRCPs were obtained by averaging segments for each condition (C, N, IC) and automatically detecting the most negative local peak at C3 (contralateral to the right hand used for button press) in the pre-response time window -200 to 0 ms [29].

EEG parameters for the C, N, and IC conditions were based, on average, on 93 trials (range 65 – 120), 92 trials (range 66 – 115), and 89 trials (range 52 – 112). The mean latency in milliseconds and mean amplitude in microvolt for each peak were then exported for statistical analysis. Mean amplitudes were calculated as the average voltage in a 40 ms window centered ± 20 ms around the peak [25, 43]. For all statistical analysis, the N1 amplitude and latency was taken from electrode O2 because the O1 and O2 measures were highly correlated and both led to the same results. In addition, the data points from the averaged waveform O2, Fz, Cz, Pz (stimulus-locked), and C3 (response-locked) were exported and used for the creation of graphics in R with the ggplot2 package [44].

#### ERP data quality

The bootstrapped standard measurement error (bSME) according to Luck et al. [45] was estimated in order to quantify the data’s quality and the precision of the ERP parameters of interest for each individual. For this purpose, preprocessed single trial data was exported from BrainVisionAnalyzer and a custom-made R script was used to obtain bSME values (10,000 bootstrapping runs) separately for each person, condition and parameter. Within this procedure, the adapted time windows were used when the search window was changed upon visual inspection (i.e., 300–600 ms instead of 350 – 650 ms). Root mean squares (RMS) were calculated to aggregate across all participants and reliability was calculated for each ERP parameter of interest as the ratio of true variance to total variance [45].

#### Statistical analysis

All statistical analyses were done using IBM SPSS 29 (IBM Corp., Armonk, NY, USA). For each ERP parameter (N1 amplitude, N1 latency, frontal N2 amplitude, frontal N2 latency, central N2 amplitude, central N2 latency, P3 amplitude, P3 latency, MRCP amplitude, and MRCP latency), a repeated measure analysis of variance (rmANOVA) with one factor (Condition: C, N, or IC) was run in order to test for the effects of perceptual and response conflict on amplitude and latency. The Greenhouse-Geisser correction was applied to correct for violations of sphericity when necessary. Significant results were followed up by pairwise comparisons of estimated marginal means.

For mediation analysis, SPSS PROCESS macro v4.0 was used [46]. A parallel mediation model was used to study the direct effect (*c’*) of fitness (measured with TMST) on flanker task performance (measured as RT) as well as the indirect effect (*ab*) mediated through the ERP parameters: N1 amplitude, N1 latency, frontal N2 amplitude, frontal N2 latency, central N2 amplitude, central N2 latency, P3 amplitude, P3 latency, MRCP amplitude, and MRCP latency. In addition, the total effect (*c*) was estimated in order to quantify the relationship between fitness and flanker performance without controlling for the mediators. Sex was also included as a covariate in the model. Figure s1 in the Supplement includes a graphical illustration of the full model. All regression coefficients were reported as standardized coefficients and effects were regarded as significant at *p*-values < 0.05. Bootstrapped (5,000 runs) confidence intervals were used to determine the significance of indirect effects. Three separate mediation models were analyzed, one for each condition of the task. Only significant results are reported in this text. For ease of understanding, the illustrations of the results were pruned to display the mediators that revealed significant results. Tables s1–s3 in the Supplement include the full model statistics.

## Results

### ERP data quality as indicated by bSME and reliability

The data quality (Table 3) resulting from our experimental protocol and preprocessing pipeline was comparable to other studies reporting the bSME [45, 47]. The reliability coefficients ranged from 0.49 to 0.98. All amplitude measures (range 0.88–0.99) and latencies of the N1 and P3 peaks (range 0.77–0.89) had adequate to good reliability. For all parameters, the mean amplitude was more reliable than the corresponding latency parameter. However, the latency measures for the central and frontal N2 peak (range 0.58–0.73) and MRCP latency (range 0.49–0.63) were found to have poor reliability, limiting the interpretation of these results.

**Table 3:** Data quality and reliability for each ERP parameter of interest

Measure	Congruent		Neutral		Incongruent	
	*RMS*	*Rel*	*RMS*	*Rel*	*RMS*	*Rel*
N1 Amplitude	0.5	0.99	0.5	0.99	0.6	0.99
N1 Latency	8.3	0.88	7.6	0.89	7.5	0.90
N2 Cz Amplitude	0.4	0.93	0.4	0.92	0.4	0.92
N2 Cz Latency	21.8	0.73	26.2	0.66	23.2	0.69
N2 Fz Amplitude	0.4	0.95	0.4	0.95	0.4	0.94
N2 Fz Latency	19.7	0.58	20.2	0.66	21.1	0.69
P3 Amplitude	0.4	0.92	0.4	0.91	0.4	0.88
P3 Latency	37.2	0.83	38.4	0.79	45.6	0.77
MRCP Amplitude	0.3	0.89	0.3	0.91	0.3	0.77
MRCP Latency	30.6	0.63	31.0	0.59	34.0	0.49

*RMS* root mean square of the bootstrapped standardized measurement error, *Rel* reliability

### ERP condition effects

**Fig. 2:**
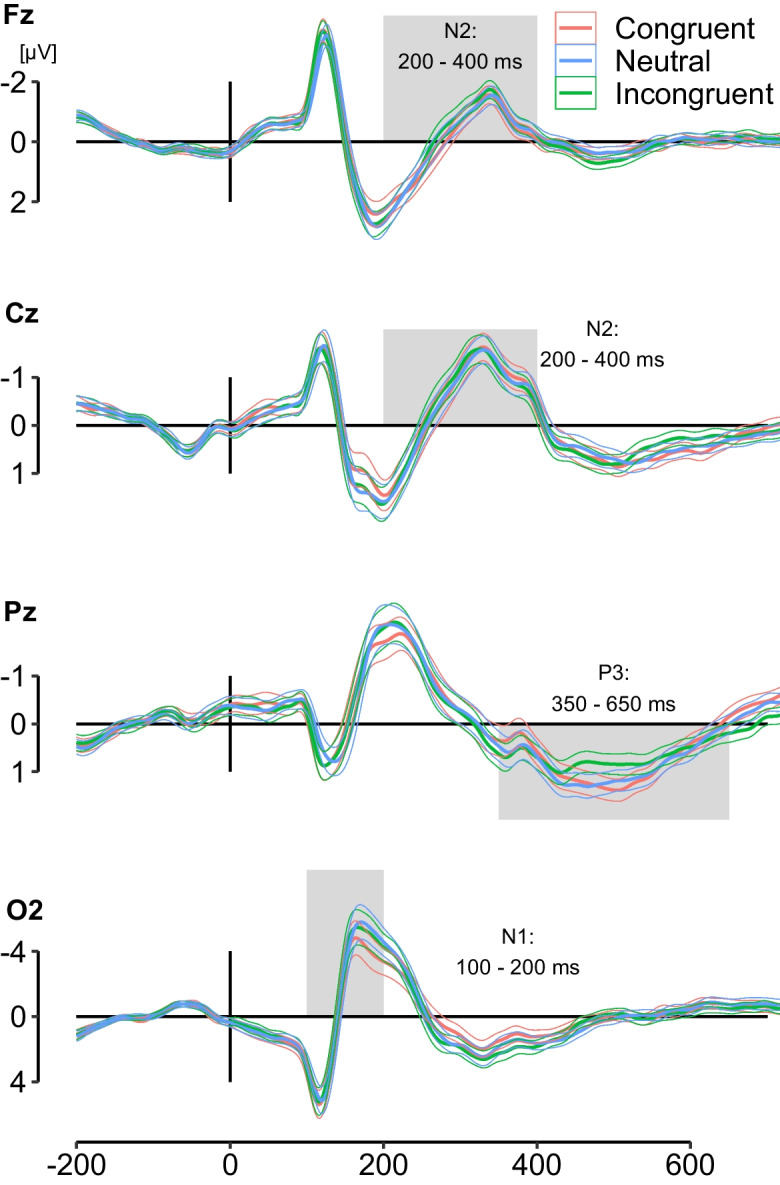
Grand averages of stimulus-locked ERPs. Thin lines depict 95% confidence intervals

**Fig. 3:**
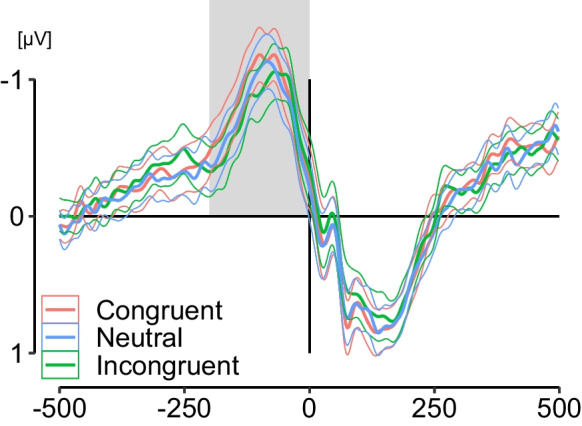
Grand average of the response-locked motor-related cortical potential at C3. Thin lines depict 95% confidence intervals

The stimulus-locked grand averages of O2, Pz, Cz, and Fz (Fig. 2) and the response-locked grand averages of C3 (Fig. 3) show the peaks (N1, N2, P3, and MRCP) of interest and indicate that the manipulation of distraction by different flankers was successful. The rmANOVA results revealed that the N1 (amplitude: *η*^2^ = 0.34, latency: *η*^2^ = 0.08), frontal N2 (amplitude: *η*^2^ = 0.07), P3 (amplitude: *η*^2^ = 0.09) and MRCP (amplitude: *η*^2^ = 0.04) differed significantly between conditions (Table 4). The N1 amplitude significantly differed between all three conditions with the N condition having the largest amplitude and the C condition having the smallest amplitude. In addition, the peak occurred later in the N trials than in the C and IC trials. The frontal N2 amplitude was larger in the IC trials and the P3 amplitude was smaller in the IC trials than in the C and N trials: This has previously been interpreted as an indicator of response inhibition [27]. The pairwise comparisons for the MRCP amplitude revealed that the amplitude was smaller for the IC condition than the C condition.

**Table 4:** Average ERP latency and amplitude and ANOVA results for the condition effects on these parameters

Measure	Congruent		Neutral		Incongruent				Pairwise
	*M*	*SD*	*M*	*SD*	*M*	*SD*	*F*	*η*^2^	test
N1 Latency	172	24	177	23	172	23	9.47***	.08	C/IC < N
N1 Amplitude	-5.5	4.7	-6.6	4.8	-6.3	4.9	59.43***	.34	N < IC < C
N2 Cz Latency	328	42	327	45	321	42	3.04	.03	
N2 Cz Amplitude	-1.9	1.5	-1.8	1.4	-1.8	1.5	0.62	.01	
N2 Fz Latency	333	30	331	34	331	38	0.41	.00	
N2 Fz Amplitude	-1.6	1.6	-1.6	1.6	-1.8	1.5	8.66***	.07	C/N > IC
P3 Latency	474	90	471	83	463	96	1.85	.02	
P3 Amplitude	2.2	1.3	2.2	1.2	2.0	1.1	11.18***	.09	C/N < IC
MRCP Latency	-92	48	-86	46	-87	47	1.21	.01	
MRCP Amplitude	-1.5	1.0	-1.4	1.1	-1.4	1.0	4.80**	.04	C < IC

Amplitude was reported in microvolt and latency in milliseconds. All tests were calculated with F(1,114) uncorrected degrees of freedom and pairwise comparisons were Bonferroni corrected

^***^ *p* < .001 ** *p* < .01 * *p* < .05

### Mediation analysis results

In each condition, the total effect of the mediation model revealed that fitness was significantly related to flanker performance (C: *c* = −0.30,* p* = 0.002; N: *c* = −0.33, *p* < 0.001; IC: *c* = −0.33, *p* < 0.001). The direct effect of fitness was significant but smaller than the total effect (C: *c’* = −0.22, *p* = 0.018; N: *c’* = -0.29, *p* = 0.002; IC: *c’* = −0.31, *p* = 0.001). This means that participants had shorter RTs for more steps in the fitness test.

**Fig. 4:**
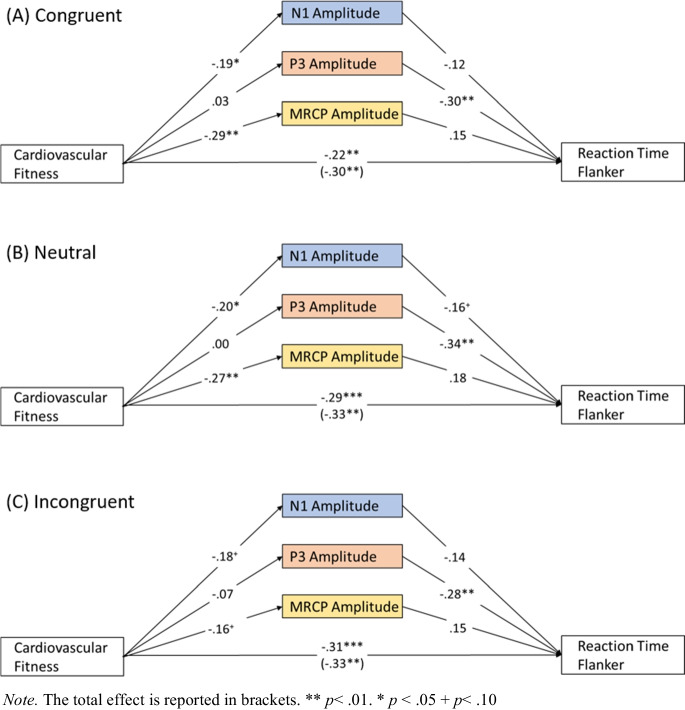
Results of the mediation analysis are presented as standardized coefficients. The total effect is reported in brackets and the direct effect above it. The mediators depicted were related to early visual processing (blue), cognitive control processes (red), and motor response generation (yellow)

For the stimulus-locked ERPs, no significant indirect effects emerged in any of the three mediation models (Fig. 4). Better fitness was associated with larger N1 amplitudes (C: *a* = −0.19, *p* = 0.049, N: *a* = −0.20, *a* = 0.035, IC: *a* = −0.18, *p* = 0.055). The only ERP parameter related to flanker performance was the P3 amplitude and larger amplitudes were associated with faster RTs (C: *b* = −0.30, *p* = 0.001, N: *b* = −0.34, *p* = 0.002, IC: *b* = −0.28, *p* = 0.008) in all conditions (Fig. 4). Once again, for response-locked potentials, no significant indirect effect of fitness, mediated through MRCP characteristics, was revealed. The MRCP amplitude was larger in people with better cardiorespiratory fitness (C: *a* = −0.29, *p* = 0.003, N: *a* = −0.27, *p* = 0.004, IC: *a* = −0.16, *p* = 0.095). No significant effects were found for the latency of the MRCP were revealed. Beyond the effects of interest, the inclusion of the covariate sex showed that females had significantly earlier occipital N1 peaks and later parietal P3 peaks (all conditions). Significant amplitude differences were only present in the IC condition for which women displayed smaller frontal N2 peaks.

## Discussion

We used the flanker paradigm to measure the relationship between cardiorespiratory fitness and EF in a sample of 80 + -year-olds. In addition, mediation models were used to determine whether the effects of fitness were mediated through electrocortical potentials related to visual processing (N1), cognitive control (N2 and P3) and motor response generation (MRCP). The positive effect of cardiorespiratory fitness on cognitive performance, as indicated by quicker responses, was replicated in this sample of 80 + -year-olds. Therefore, cardiorespiratory fitness seems to be a promising starting point for lifestyle interventions, even in very old age. Studying these effects in more detail revealed that the relationship between fitness and RT was similar in all three conditions of the flanker task with fitness being related to larger occipital N1 and MRCP amplitudes. Hence, there was no specific effect of fitness on the resolution of the visual conflict during N trials or response conflict during IC trials. This was also supported by the finding that fitness was not significantly related to later cognitive control processes such as attentional control and response inhibition measured by the N2 and P3 components.

### Fitness is related to early visual processing (N1) but not later processing (N2, P3)

In our sample, fitness was significantly associated with improved early visual processing as indicated by increased occipital N1 amplitudes in people with better cardiorespiratory fitness. This finding is supported by another study which reported that more physically active OA had larger N1 amplitudes when compared to their less active counterparts [12]. The N1 amplitude can be interpreted as evidence that the subject has more resources available for early visual processing.

The N2 has been shown to be sensitive to the effects of physical activity in attentional control tasks including the flanker task [11]. This finding was not replicated here as no significant relationship between fronto-central N2 amplitude or latency and fitness was revealed. This deviation could be related to different analytical strategies applied on the flanker outcomes. Winneke et al. [11] used the difference between conditions (N – C and IC – C) as a measure of conflict resolution rather than absolute values in one condition. As the flanker is designed to facilitate the investigation of such differences, we replicated this approach but still found no significant relationship between fitness and RT differences (see Supplement Tables s4–s5). Therefore, we opted to run separate analyses for each condition in order to be able to find effects that might be unrelated to conflict resolution. In addition,  a meta-regression has shown that the effect of physical activity was only partially mediated through fitness [48]. Hence, the differing results obtained by Winneke et al. [11] might be related to using self-reported physical activity as the variable of interest instead of objectively measured cardiorespiratory fitness.

While P3 showed modulation depending on the type of condition and P3 amplitude was associated with RT, there was no significant relationship to cardiorespiratory fitness in our sample. This is in contrast to the findings of a meta-analysis which reported an association between fitness and larger amplitudes and shorter latency for P3 [19]. Only four studies included in the meta-analysis examined the relationship in OA and none of them included participants as old as the SENDA sample, all used small sample sizes of 30 or less participants. In addition, instead of treating fitness as a continuous measure, the subjects were split into groups of high and low fitness, which probably explains the differing results. It has also been shown that cognitive status is a relevant moderator of the association between fitness and P3 amplitude as fitness was correlated with P3 amplitude in a group of OA with amnestic MCI but not in the age-matched group of healthy OA [20]. This is in accordance with our findings which revealed a non-significant relationship between P3 amplitude and fitness in cognitively healthy OA (MoCA > 23).

Taken together, our results suggest that the positive effects of cardiorespiratory fitness that have been detected may be related to changes in early visual processing rather than cognitive control processes as we hypothesized. From the supplementary analysis of the differences between conditions it became also clear that the effects do not seem to be specific to a certain condition of the flanker task. Meaning they are not related to different modulations of ERP characteristics in respect to the presence of perceptual conflict (N – C) or an additional response conflict (IC – C). This challenges prior findings postulating that EF are specifically sensitive to the effects of chronic physical activity and more strongly related to fitness than other cognitive functions [9]. All the above points have demonstrated that a detailed analysis of cortical processes is essential when interpreting the behavioral results found in EF tasks such as the flanker.

### Fitness is related to response-locked motor related cortical potentials

The MRCP obtained through response-locking was related to cardiorespiratory fitness in the OA. Here, better fitness was related to larger MRCP amplitudes in all three conditions. This could be interpreted as an indication that more neural resources were available for motor planning and response generation in the fitter OA. Our results on this point align with a study that compared older and younger adults and found that OA reacted more slowly and had larger MRCP amplitudes. This was then interpreted as evidence that a higher activation level was needed for successful task execution in OA [30]. The higher activation found in older adults (compared to younger adults) when successfully performing a task is often considered to be the result of a neural compensation process and it has been shown that the hypoactivation of the sensorimotor regions specifically is correlated with declined performance in motor control tasks in OA [49]. Hence, we speculated that larger MRCP amplitudes in fitter OA can be interpreted as evidence of successful neural compensation. Although the MRCP amplitude was related to fitness, it could not be shown that the differences in MRCP amplitude mediated the fitness effect found in flanker performance. One reason for the missing link between MRCP amplitude and flanker performance might be the timely overlap of the P3 component and the MRCP, which might have masked effects [50]. In the future, it could, potentially, be helpful to examine the differences in ERPs between fast and slower RTs by binning according to RT [51]. We have refrained from such analysis here because it requires a larger number of trials than was available for each of our participants.

There was no relationship between the latency of MRCPs and fitness or flanker performance. The latency has previously been interpreted as the endpoint of the deliberation process in a choice reaction task [31, 32]. This would mean that the length of the deliberation process is not dependent upon the fitness level of the participants. Alternatively, this non-finding could also be related to the poor reliability of the MRCP latency measure.

### Potential biological mechanisms

In our study, a higher level of cardiorespiratory fitness was related to higher neuronal activation as indicated by larger amplitudes (N1 and MRCP). Different mechanisms have been proposed to explain the higher brain activation observed in fitter individuals [9]. In addition to other factors, reduced grey matter loss has been reported in fitter OA [52] and this has been interpreted as an indication of the increased availability of neuronal resources. Region-specific effects of fitness and exercise have been described for grey matter volume, indicating a positive relationship between grey matter volume and cardiorespiratory fitness, specifically in the prefrontal cortex and hippocampus [9]. However, the findings of the current study do not support this observation because the effects of fitness were found in different cortex areas related to early visual processing (occipital cortex) and motor response generation (i.e., sensorimotor cortex).

 Cardiorespiratory fitness can be considered as a proxy indicator for being physically active, which requires quickly processing visual input and producing motor output very regularly. Other processing components related to cognitive control (as captured by N2 and P3) could be well used and maintained by OA through leisure activities, e.g., cross words, artistic activities, reading, rather than physical activities. Hence, it seems that, particularly in the oldest old, the specific levels of processing that associated with being physically active are related to cardiorespiratory fitness. This might be particularly prominent in this sample as, in the age range of our sample (aged 80 years and older), “using it” is even more important for maintaining neuronal networks than it is in younger samples.

### Limitations

Our study has several limitations. We assessed cardiorespiratory fitness using the TMST while most other studies use VO2 peak measurements as an indicator of cardiorespiratory fitness [15, 19]. The TMST might not only be a measure of cardiorespiratory fitness alone, it may also measure balance and orthopedic restriction, which are common in this age group. Therefore, the results obtained might be related to these factors rather than cardiorespiratory fitness alone. Despite this, this test was chosen over the alternatives because of its age appropriateness [37], established validity and responsiveness [38], and correlation with the VO2 peak [53] and because it has been shown that participants older than 70 years are not able to achieve maximal effort during ergometer testing [54].

When interpreting our results, it must also be considered that the study was only cross-sectional and, hence, the direction of effects is not clear. For example, it might be that people with better cognitive performance are able and inclined to live more active daily lives and that better overall fitness is the result, not the cause. Nevertheless, by investigating the mediating effects of the EEG parameter the findings of this study can be considered a meaningful addition to our understanding of the relationship between fitness and cognition in the old-old age group, specifically.

### Summary

Even in OA (≥ 80 years old) there was a positive relationship between cardiorespiratory fitness and cognition when using the flanker task. The flanker task is designed to measure EF such as attentional control and response inhibition. However, using only behavioral outcomes (like RT) limits the ability to determine the underlying causes of the improvements in behavior. To the best of our knowledge, this is the first study to use mediation analysis to understand at which levels of processing fitness effects can be found in old-old adults. The results of our study suggest that the faster performance in the flanker task might be related to more neuronal resources for motor planning and preparation in fitter OA, while cognitive control processes were not significantly associated with fitness in our sample. In addition, early visual processing also seemed to be improved in fitter OA. Any future ERP study focusing on the effects of cardiorespiratory fitness, physical activity, and/or exercise in OA should include measures related to motor response generation when studying EF using choice reaction tasks in order to further detangle the association of fitness with different processing levels.

Based on our findings, we assume that fitter older adults probably perform many cognitive tasks, not only tasks related to EF, quicker because they have more resources available for early visual processing and motor response generation. While we can make no prediction from our results on whether improving cardiorespiratory fitness in old-old age would have a positive effect, it demonstrates the importance of maintaining fitness into old age because the current fitness status of 80 + -year-olds is positively associated with cognition and neuronal resources. However, this may also mean that older adults with poor fitness might have an increased risk of cognitive deficits. Considering the growing number of OA and the lack of resources in the health and elderly care sectors, our study supports the importance of an active lifestyle and upkeeping cardiorespiratory fitness for independence until old age.

### Supplementary Information

Below is the link to the electronic supplementary material.

Supplementary file1 (DOCX 98 KB)

## Data Availability

The raw data supporting the conclusion of this article will be made available by the authors. Request to access the datasets should be directed to Claudia Voelcker-Rehage (claudia.voelcker-rehage@uni-muenster.de).
