# Supplementary material for: Cardiorespiratory fitness is associated with cognitive performance in 80 + -year-olds: Detangling processing levels
Source: GeroScience. 2024 Jan 23;46(3):3297–310. doi: 10.1007/s11357-024-01065-8 (PMC11009210; doi:10.1007/s11357-024-01065-8)
Supplement: Supplementary file 1 — Supplementary file1 (DOCX 98 KB) [file 11357_2024_1065_MOESM1_ESM.docx]

**Supplementary Material**

Provided for “Cardiorespiratory fitness is associated with cognitive performance in 80+-year-olds: detangling processing levels” by Fröhlich, Kutz, Müller and Voelcker-Rehage

Fig s1.

Parallel mediation model with all mediators of interest.


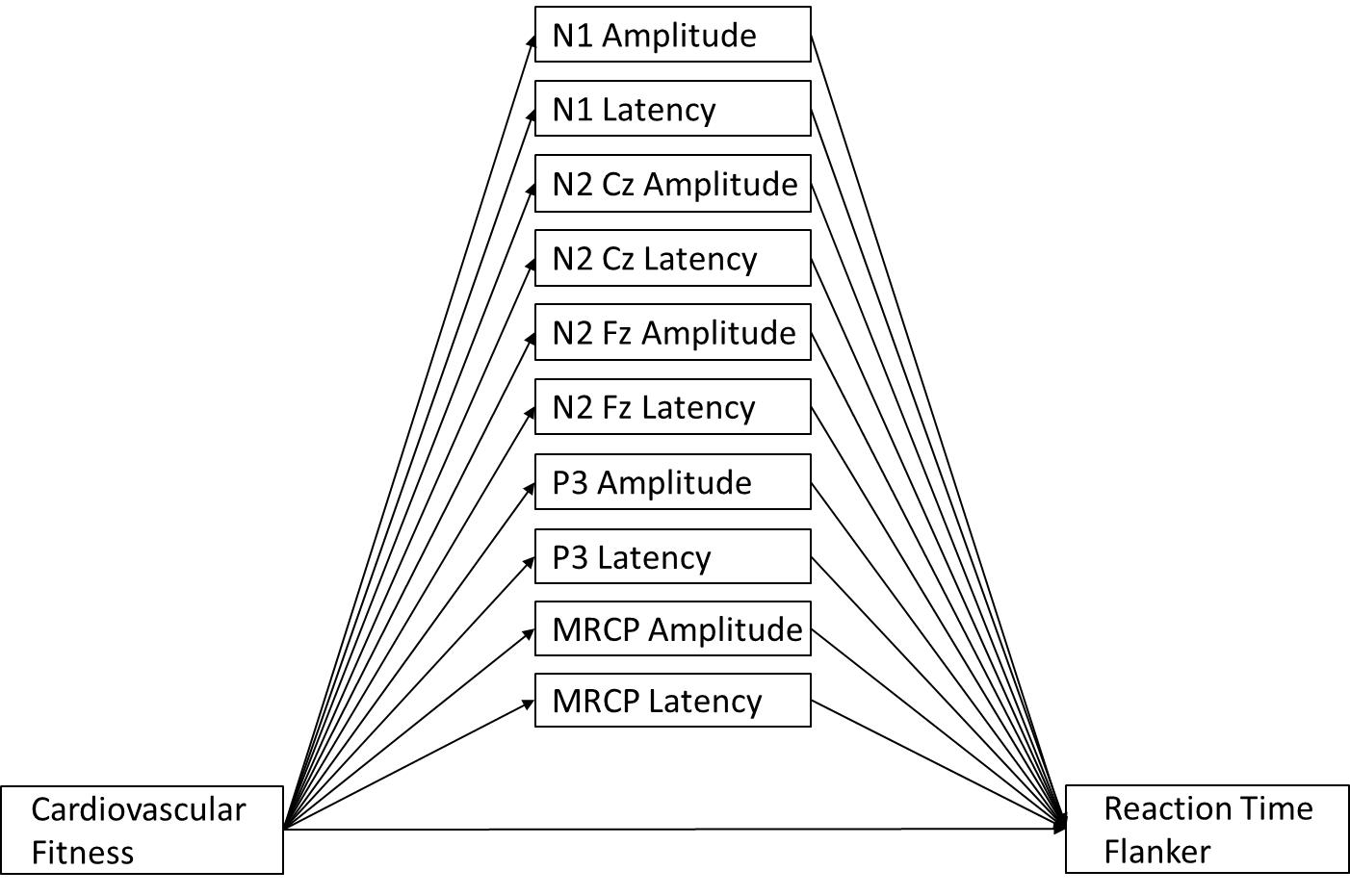


*Note.* Covariate sex was included into the model.

Tab s1. Results of the parallel mediation analysis with the dependent variable mean reaction time of congruent flanker trials

| Mediator | Effect IV on mediator (a) | Unique effect of mediator (b) | Indirect Effect (ab) | Bootstrapped 95% CI | |
| --- | --- | --- | --- | --- | --- |
|  |  |  |  | Lower | Upper |
| N1 Latency | .02 | .10 | .00 | -.03 | .05 |
| N1 Ampl | **-.19*** | .07 | .00 | -.05 | .03 |
| N2 Cz Latency | -.06 | .00 | -.00 | -.04 | .02 |
| N2 Cz Ampl | -.15 | -.00 | .00 | -.04 | .05 |
| N2 Fz Latency | -.10 | .17^+^ | -.02 | -.05 | .02 |
| N2 Fz Ampl | .08 | -.13 | -.01 | -.05 | .02 |
| P3 Latency | .13 | -.10 | -.01 | -.06 | .02 |
| P3 Ampl | .03 | **-.30**** | -.01 | -.07 | .05 |
| MRCP Latency | .01 | .01 | .00 | -.02 | .02 |
| MRCP Ampl | **-.29**** | .15 | -.04 | -.14 | .02 |
|  |  |  |  |  |  |
| Total effect | **-.30**** |  |  |  |  |
| Direct Effect | **-.22*** |  |  |  |  |

Note. All effects reported are standardized coefficients. This model also included the covariate sex. IV = Independent variable was number of steps in two minute step test, Ampl = Amplitude.

*** p < .001 ** p <.01 * p <.05 ^+^ p <.10

Tab s2. Results of the parallel mediation analysis with the dependent variable mean reaction time of neutral flanker trials

| Mediator | Effect IV on mediator (a) | Unique effect of mediator (b) | Indirect Effect (ab) | Bootstrapped 95% CI | |
| --- | --- | --- | --- | --- | --- |
|  |  |  |  | Lower | Upper |
| N1 Latency | -.05 | .10 | -.01 | -.05 | .02 |
| N1 Ampl | **-.20*** | -.16^+^ | .03 | -.00 | .10 |
| N2 Cz Latency | .06 | -.13 | -.01 | -.05 | .02 |
| N2 Cz Ampl | -.14 | -.01 | .00 | -.04 | .05 |
| N2 Fz Latency | -.06 | .08 | -.00 | -.03 | .02 |
| N2 Fz Ampl | .08 | -.13 | -.01 | -.06 | .02 |
| P3 Latency | .11 | -.06 | -.01 | -.04 | .02 |
| P3 Ampl | -.02 | **-.34**** | .01 | -.06 | .08 |
| MRCP Latency | .06 | .10 | .01 | -.02 | .04 |
| MRCP Ampl | **-.27**** | .18 | -.05 | -.14 | .02 |
|  |  |  |  |  |  |
| Total Effect | **-.33***** |  |  |  |  |
| Direct Effect | **-.29**** |  |  |  |  |

Note. All effects reported are standardized coefficients. This model also included the covariate sex. IV = Independent variable was number of steps in two minute step test, Ampl = Amplitude.

*** p < .001 ** p <.01 * p <.05 ^+^ p <.10

Tab s3. Results of the parallel mediation analysis with the dependent variable mean reaction time of incongruent flanker trials

| Mediator | Effect IV on mediator (a) | Unique effect of mediator (b) | Indirect Effect (ab) | Bootstrapped 95% CI | |
| --- | --- | --- | --- | --- | --- |
|  |  |  |  | Lower | Upper |
| N1 Latency | -.02 | .16+ | -.00 | -.05 | .03 |
| N1 Ampl | **-**.18^+^ | -.14 | .03 | -.01 | .09 |
| N2 Cz Latency | -.02 | -.04 | .00 | -.03 | .03 |
| N2 Cz Ampl | -.08 | .02 | -.00 | -.03 | .03 |
| N2 Fz Latency | -.09 | .03 | -.00 | -.04 | .02 |
| N2 Fz Ampl | .14 | -.19 | -.03 | -.09 | .02 |
| P3 Latency | .14 | -.03 | -.00 | -.04 | .04 |
| P3 Ampl | -.07 | **-.28**** | .02 | -.04 | .09 |
| MRCP Latency | -.02 | -.06 | .00 | -.02 | .02 |
| MRCP Ampl | -.16+ | .15 | -.02 | -.09 | .01 |
|  |  |  |  |  |  |
| Total effect | **-.33**** |  |  |  |  |
| Direct Effect | **-.31**** |  |  |  |  |

Note. All effects reported are standardized coefficients. This model also included the covariate sex. IV = Independent variable was number of steps in two minute step test, Ampl = Amplitude.

*** p < .001 ** p <.01 * p <.05 ^+^ p <.10

Tab s4. Results of the parallel mediation analysis with the dependent variable difference of mean reaction times IC – C

| Mediator | Effect IV on mediator (a) | Unique effect of mediator (b) | Indirect Effect (ab) | Bootstrapped 95% CI | |
| --- | --- | --- | --- | --- | --- |
|  |  |  |  | Lower | Upper |
| N1 Latency | -.08 | .08 | -.01 | -.05 | .03 |
| N1 Ampl | -.02 | .09 | -.00 | -.03 | .03 |
| N2 Cz Latency | .04 | -.12 | -.01 | -.05 | .02 |
| N2 Cz Ampl | .15 | .11 | .02 | -.02 | .06 |
| N2 Fz Latency | -.01 | -.07 | .00 | -.02 | .02 |
| N2 Fz Ampl | .11 | -.10 | -.01 | -.05 | .01 |
| P3 Latency | .01 | .08 | .00 | -.02 | .03 |
| P3 Ampl | -.17^+^ | -.12 | .02 | -.02 | .06 |
| MRCP Latency | -.03 | .10 | -.00 | -.04 | .02 |
| MRCP Ampl | **.23*** | .01 | .00 | -.06 | .05 |
|  |  |  |  |  |  |
| Total effect | -.12 |  |  |  |  |
| Direct effect | -.13 |  |  |  |  |

Note. All effects reported are standardized coefficients. This model also included the covariate sex. IV = Independent variable was number of steps in two minute step test, Ampl = Amplitude.

*** p < .001 ** p <.01 * p <.05 ^+^ p <.10

Tab s5. Results of the parallel mediation analysis with the dependent variable difference of mean reaction times N – C

| Mediator | Effect IV on mediator (a) | Unique effect of mediator (b) | Indirect Effect (ab) | Bootstrapped 95% CI | |
| --- | --- | --- | --- | --- | --- |
|  |  |  |  | Lower | Upper |
| N1 Latency | -.15 | .11 | -.02 | -.07 | .01 |
| N1 Ampl | -.09 | .01 | -.00 | -.03 | .03 |
| N2 Cz Latency | .15 | -.01 | -.00 | -.04 | .03 |
| N2 Cz Ampl | .04 | .14 | .01 | -.02 | .04 |
| N2 Fz Latency | .03 | -.16^+^ | -.01 | -.04 | .04 |
| N2 Fz Ampl | -.01 | .13 | -.00 | -.03 | .03 |
| P3 Latency | -.04 | .11 | -.00 | -.03 | .02 |
| P3 Ampl | .09 | -.16^+^ | .01 | -.03 | .06 |
| MRCP Latency | .05 | .07 | .00 | -.02 | .04 |
| MRCP Ampl | -.01 | .09 | .00 | -.04 | .02 |
|  |  |  |  |  |  |
| Total effect | -.11 |  |  |  |  |
| Direct effect | -.11 |  |  |  |  |

Note. All effects reported are standardized coefficients. This model also included the covariate sex. IV = Independent variable was number of steps in two minute step test, Ampl = Amplitude.

*** p < .001 ** p <.01 * p <.05 ^+^ p <.10
